# Risk of malignancy and the use of disease-modifying therapy in multiple sclerosis: exploring the role of DMT in a multi-center study

**DOI:** 10.3389/fneur.2024.1492678

**Published:** 2024-12-13

**Authors:** Mohammed Bayounis, Mohammed Bin Mahfooz, Mohammed Assiri, Yousef Alghamdi, Fahad Alturki, Mahdi Almutairi, Abdullah Alshelawy, Mohammed Alosaimi, Faisal Almutawa, Abdulrahman Alammar, Yahya Alyahya, Osama Khojah, Seraj Makkawi, Yaser Al Malik

**Affiliations:** ^1^College of Medicine, King Saud Bin Abdulaziz University for Health Sciences, Riyadh, Saudi Arabia; ^2^King Abdullah International Medical Research Center, Riyadh, Saudi Arabia; ^3^College of Medicine, King Saud Bin Abdulaziz University for Health Sciences, Jeddah, Saudi Arabia; ^4^King Abdullah International Medical Research Center, Jeddah, Saudi Arabia; ^5^Department of Neuroscience, Ministry of the National Guard-Health Affairs, Jeddah, Saudi Arabia; ^6^Department of Neurology, Ministry of the National Guard-Health Affairs, Riyadh, Saudi Arabia

**Keywords:** multiple scleorsis, neuroimmunology, malignancy, disease modifying therapies, DMT

## Abstract

**Background:**

The potential link between disease-modifying therapies (DMTs) and malignancy in multiple sclerosis (MS) patients has generated significant concern, particularly given the immunosuppressive nature of these treatments. Conflicting evidence in the literature has left this issue unresolved, underscoring the need for definitive research to inform clinical practice. This study addresses this gap by examining cancer occurrence among MS patients on DMTs treated at two tertiary-care centers in Saudi Arabia.

**Objectives:**

To report and analyze cases of malignancy in MS patients treated with DMTs and identify associated risk factors, including demographic characteristics, Expanded Disability Status Scale (EDSS) scores, treatment duration, and cumulative DMT exposure. The study also seeks to contribute to the development of evidence-based cancer screening protocols for MS patients at elevated risk.

**Methods:**

A retrospective review was performed on medical records of MS patients treated with DMTs at two tertiary-care centers in Saudi Arabia from June 2015 to December 2023. The study included 860 patients, with data collected on demographics, MS subtype, DMT usage, and subsequent cancer diagnoses. A comprehensive literature review, covering publications from February 1976 to May 2024, supplemented the review to contextualize findings within the broader research landscape.

**Results:**

Among the 860 MS patients on DMTs, 10 (1.16%) developed malignancies, predominantly female (80%), with an average age of 50.9 years. The majority had relapsing–remitting MS (RRMS) (90%), with interferon beta (70%) and ocrelizumab (60%) being the most frequently used DMTs. The median duration of DMT exposure prior to cancer diagnosis was 52 months. The observed malignancies included gynecological cancers, breast cancer, thyroid cancer, and nasopharyngeal carcinoma, with 60% diagnosed at advanced stages (III-IV).

**Conclusion:**

This case series highlights instances of malignancy among MS patients undergoing DMTs, suggesting a potential link that warrants further investigation. The findings underscore the need for vigilant cancer screening and patient education as integral components of MS management. The literature review reinforces the necessity for ongoing research to better understand the risks associated with DMTs, aiding in the development of more informed clinical guidelines. Further large-scale, longitudinal studies are crucial to elucidate the causality and guide safer treatment strategies.

## Introduction

Multiple sclerosis (MS) is a chronic non-traumatic degenerative disease of the central nervous system that is hallmarked by demyelination and autoimmune inflammation. It is a major contributor to neurological disability in young adults and affects women nearly three times more often than men, with the highest incidence occurring between the ages of 20 and 45 (1). The pathogenesis mainly involves T-cell dysfunction that leads to axonal damage and the formation of acute plaques. B-lymphocytes have also been implicated in the process; however, the exact mechanism is still unclear (2). Clinical features most often include optic neuritis, cranial nerve dysfunction, and spinal cord degenerative symptoms (paresthesia, motor weakness, loss of vibration, and ataxia) (3). Diagnosis is made based on the McDonald criteria, which uses imaging, CSF analysis, and clinical presentation to categorize symptoms based on dissemination in space and dissemination in time (4). Treatment for the disease involves immunosuppression, either acutely in flares using steroids, or using disease-modifying therapy (DMT) long term (5). There have been numerous advances within the last two decades in DMT options. Interferons and glatiramer acetate have been used since the turn of the century with modest results yet excelling in their safety profile. Over time, increasingly targeted therapies have been utilized, like natalizumab, a monoclonal antibody that inhibits lymphocyte migration into the central nervous system, which has been shown to have a powerful effect with excellent results (6). Other drugs recently approved include cladribine and teriflunomide, which are DNA synthesis inhibitors, and fingolimod, which is part of the sphingosine 1-phosphate receptor modulators (7, 8). Lymphocyte depletion agents like ocrelizumab, alemtuzumab, and the off-label use of rituximab have been widely used and have favorable outcomes (9).

The efficacy of these drugs has been well-established in the literature; however, the specific cancer-related adverse events have been studied less extensively and require more large and long term prospective trials. Some drugs have established adverse events, like natalizumab and the occurrence of progressive multifocal leukoencephalopathy. DMTs have also been notably linked to opportunistic infections (10). Due to its association with immune suppression, secondary malignancies are also a concerning side effect. Safety concerns has been raised due to the increase in incidence of cancer in patients on immunosuppressive therapy, particularly in MS patients (11, 12). With the conflicting results in the literature, there is no agreeing consensus on the risk of developing cancer, which is important to establish so that we can implement screening protocols for at-risk patients and decrease morbidity and mortality burden.

Our study aims to report cancer development in MS patients who use DMTs, and showcase related risk factors such as demographic data, relations with expanded disability status scale (EDSS), treatment duration, and cumulative doses.

## Materials and methods

A retrospective review of medical records of all patients with a diagnosis of MS between June 2015 to December 2023 was performed. This study was conducted across two tertiary-care centers King Abdulaziz Medical City (KAMC) in Riyadh and Jeddah, Saudi Arabia. All patients with a diagnosis of MS and taking DMTs who then developed a malignancy were included in this study. A non-probability consecutive sampling method was utilized for participant selection. Data from the hospital’s electronic medical record system were collected and utilized for a thorough analysis. Information collected included demographics, MS clinical characteristics, treatments and their duration, and cancer development. Institutional review board (IRB) approval was obtained from King Abdullah International Medical Research Centre (KAIMRC) (Protocol Approval Number NRC23R/378/05).

A thorough literature review was also conducted. A PubMed search identifying original studies using the terms “multiple sclerosis,” “disease modifying therapy,” and “malignancy” OR “cancer” was done in May 2023. Articles published between February 1976 and May 2024 were searched. We excluded case reports, case series, and reviews from our search. Published work that is not in English were also excluded.

## Results

In this retrospective study of MS patients treated with DMTs who subsequently developed cancer, we reviewed data from 860 patients in both centers collectively and 10 cases were identified and met the inclusion criteria (1.16%) as seen in Table 1. The gender distribution was predominantly female (80%) compared to male (20%). The mean age of the patients was 50.9 ± 8.7 years. Relapsing–remitting MS (RRMS) was the predominant type of MS (90%), with only one case of secondary progressive MS (SPMS) (10%). The DMTs used among these patients were interferon beta, ocrelizumab, fingolimod, teriflunomide, and ofatumumab. The frequencies of DMT usage were as follows: interferon beta was used in 7/10 patients (70%). Ocrelizumab usage accounted for 6/10 patients (60%). Both fingolimod and teriflunomide were used in two cases (20% each), while only one case was found to be on ofatumumab (10%). Five cases (50%) were treated with 2 or more DMTs prior the development of malignancy, all of whom were treated with interferon beta at some point and expressed different types of malignancy, as seen in the table. The duration of DMT usage until the development of cancer had a median of 52 months, with the shortest being 4 months (case 7) and the longest being almost 360 months (30 years; case 8). When excluding cases with missing data (cases 8 and 9), the median duration was 30 months. Out of the 10 cases, 6 had late stage (stage 3–4) malignancies, but only one patient (case 9) had developed metastasis. Regarding the EDSS at the time of cancer diagnosis, it had a mean score of 4.0 ± 2.49, with the lowest score being 0 and highest being 7.5. All patients were alive at last follow-up.

**Table 1:** Demographics of MS patients on DMT and their cancer-related outcomes.

Case	Gender	Age	Year of MS diagnosis	Type of MS	DMT	Total treatment duration (months)	Type of cancer	Year of cancer diagnosis	Stage	Metastasis	EDSS on cancer diagnosis	Survival on last follow-up
Case 1	Male	47	2014	RRMS	Interferon beta-1b 44mcg for 24 mo	24	Nasopharyngeal carcinoma	2016	IVA	-	2.5	Yes
Case 2	Female	40	2007	RRMS	Interferon beta-1b 30mcg for 36 mo	36	Papillary thyroid cancer	2018	I	-	2	Yes
Case 3	Female	41	2021	RRMS	Ocrelizumab 600 mg for 13 mo	13	Infiltrating ductal carcinoma of the breast	2022	IIB	-	0	Yes
Case 4	Male	36	2009	RRMS	Ocrelizumab 600 mg for 24 mo	24	Multiple myeloma	2021	3^‡^	-	6.5	Yes
Case 5	Female	57	2008	RRMS	Interferon beta-1a 30mcg for 132 mo	161	Endometrial adenocarcinoma	2023	IA	-	6.5	Yes
					Ocrelizumab 600 mg for 29 mo							
Case 6	Female	59	2004	SPMS	Interferon beta-1a ^*^ for 96 mo	132	Squamous cell cancer of the oral cavity	2023	IV	-	7	Yes
					Ocrelizumab 600 mg for 36 mo							
Case 7	Female	59	2008	RRMS	Ocrelizumab 600 mg for 4 mo	4	Squamous cell cancer of the cervix	2022	IIIC	-	2	Yes
Case 8	Female	60	1994	RRMS	Interferon beta ^*,†^	∼360	Infiltrating ductal carcinoma of the breast	2015	I	-	3	Yes
					Interferon beta-1a ^*,†^							
					Fingolimod 0.5 mg for 5 mo							
					Teriflunomide 14 mg for 37 mo							
Case 9	Female	58	2010	RRMS	Teriflunomide 14 mg ^†^	>68	Medullary thyroid cancer	2016	IVC	Liver and lungs	3	Yes
					Interferon beta-1a 30mcg for 37 mo							
					Ocrelizumab 600 mg for 31 mo							
Case 10	Female	52	2009	RRMS	Interferon beta * for 120 mo	136	Squamous cell cancer of the cervix	2023	IV	-	7.5	Yes
					Fingolimod 0.5 mg for 12 mo							
					Ofatumumab 20 mg/0.4 mL for 4 mo							

^*^Missing dose. ^†^Missing duration of treatment. ^‡^Based on the revised international staging system (R-ISS). DMT, disease-modifying therapy; EDSS, expanded disability status scale; MS, multiple sclerosis; RRMS, relapsing–remitting multiple sclerosis; SPMS, secondary-progressive multiple sclerosis.

We identified various types of malignancies across different systems and their associated therapies. Gynecological malignancies were observed in two cases of squamous cell carcinoma of the cervix and one case of endometrial adenocarcinoma. These patients were previously treated with interferon beta, ocrelizumab, fingolimod, and ofatumumab. Additionally, two cases of infiltrating ductal carcinoma of the breast were identified, with the patients having been on various DMTs prior to cancer development. Regarding thyroid malignancies, we observed two cases: one of papillary thyroid carcinoma in a patient treated with interferon beta, and another of medullary thyroid carcinoma in a patient who had received interferon beta, teriflunomide, and ocrelizumab. One case of nasopharyngeal carcinoma that was previously on interferon treatment and one case of squamous cell carcinoma of the oral cavity who had received interferon beta and ocrelizumab. A single case of multiple myeloma was also noted in a patient treated with ocrelizumab. We also report two MS patients that developed benign tumors while on DMTs. The first was a case of cerebellopontine angle schwannoma (WHO grade 1) in a 38-year-old female that had been on interferon beta therapy for 120 months. She had SPMS and an EDSS score of 7.5 at tumor diagnosis. The second case involved a kidney angiomyolipoma in a 45-year-old male with RRMS after 6 months of interferon beta therapy, followed by 7 months of ocrelizumab therapy (after induction and one dose of management).

## Discussion

The association between DMTs for MS patients and the risk of secondary malignancies is an important concern for clinicians and patients alike. Our retrospective study provides insights into this association given the lack of consensus on DMT-associated risk of malignancies. The study also highlights several key findings and raises crucial considerations for clinical practice and future research.

Our study identified a malignancy in 1.16% MS patients receiving DMTs in our population, a figure that emphasizes the necessity of vigilant long-term monitoring. The majority of the cases were female, with a mean age of 50.9 years, and predominantly diagnosed with RRMS (90%). These demographics align with the broader MS patient population as well as previous studies (Table 2), which is typically characterized by a higher prevalence in women and an average onset in middle age. According to the cancer incidence report issued by the Saudi health council in 2020, between January 01 and December 31, 2020, the total number of newly diagnosed cancer cases reported to the Saudi Cancer Registry (SCR) was 17,631. Overall cancer was more among women than men; it affected 8,056 (45.7%) males and 9,575 (54.3%) females. A total of 14,235 cases were reported among Saudi nationals, 3,274 among non-Saudis, and 122 of unknown nationalities. The overall age-standardized incidence rate (ASR) was 74.7/100,000 in males and 92.1/100,000 in females (13).

**Table 2:** Literature review.

First author	Year of publication	Country	Study design	Patients (*N*)	Age at diagnosis (mean or median)	Female (%)	Male (%)	MS type (*N*, %)	EDSS (mean)	Medications studied	Cancer (*N*, %)	Cancer-related mortality (*N*, %)
Alping (14)	2020	Sweden	Retrospective cohort	6,136	-	70.70%	29.30%	RRMS: 6302, 84.4% SPMS: 893, 11.9% PPMS: 179, 2.4% PRMS: 100, 1.3%	Rituximab: 2.8 Fingolimod: 2.2 Natalizumab: 2.4 Together: 2.6	Rituximab, fingolimod and natalizumab	Total: 168, 2.7% BCC: 43, 25.6% CIN3: 47, 28.0% Any invasive: 78, 46.4%; Breast: 12, 7.1% Prostate: 5, 3.0% Melanoma: 10, 6% Skin (non-melanoma): 6, 3.6% Malignant lymphoma: 3, 1.8% Colon: 10, 6% Other endocrine glands and tissues: 7, 4.1% Corpus uteri: 3, 1.8% Lung and bronchus: 2, 1.2% Bladder: 2, 1.2% Anus and anal canal: 2, 1.2% Kidney: 2, 1.2% Pancreas: 2, 1.2% Testicle: 2, 1.2% Brain: 2, 1.2% Thyroid: 1, 0.6% Rectum: 1, 0.6% Liver and intrahepatic bile duct: 1, 0.6% Leukemia: 1, 0.6% Digestive organs: 1, 0.6% Cervix uteri, collum uteri, portio: 1, 0.6% Bone, joint, and cartilage in the extremities: 1, 0.6% Vagina: 1, 0.6%	-
Ragonese (17)	2017	Italy	Retrospective cohort	531	29	63.30%	36.70%	-	-	Azathioprine, mitoxantrone, cyclophosphamide^*^ and “others” (interferon beta or glatiramer acetate)	Total: 13, 2.4% Gut (colon & rectal): 3, 23.1% Brain: 1, 7.7% Breast: 3, 23.1% Ovary: 1, 7.7%% Pancreas: 1, 7.7% Lung: 1, 7.7% Leukemia: 3, 23.1%	-
Nørgaard (18)	2019	Denmark	Retrospective cohort	10,752	39.7 (median)	67.50%	32.50%	RRMS: 6129, 57% SPMS: 753, 7% PPMS: <2% Undetermined: 3656, 34%	-	-	“Any cancer” (Total): 608, 5.7% Breast: 111, 18.3% Melanoma: 53, 8.7% Skin (non-melanoma): 166, 27.4%	138, 1.3%
Gomez-Moreno (16)	2021	Spain	Prospective cohort	886	31	71%	29%	“Relapsing” MS: 886, 100%	2.13	Dimethyl-fumarate	Total: 8, 0.9% Thyroid: 3, 37.5% Breast: 2, 25% Cervical: 1, 12.5% Uterine: 1, 12.5% Bladder: 1, 12.5%	1, 12.5%
Kingwell (15)	2014	Canada	Case–control	5,146	32	75%	25%	RRMS/PRMS: 5146, 100%	-	Interferon beta	Total: 227, 4.4% Breast: 63, 36% Lung: 20, 8.8% Prostate: 12, 23% Colorectal: 18, 7.9%	-
Mariottini (19)	2022	Italy	Retrospective cohort	661	37 (median)	68%	32%	RRMS: 574, 87% SPMS: 73, 11% PPMS: 14, 2%	PPMS 5.4 RRMS 1.7 SPMS 6.2 Together: 2.7 (median)	Glatiramer acetate, interferon-b 1A &1b, dimethyl fumarate, pegylated interferon beta-1A, teriflunomide, fingolimod, natalizumab, alemtuzumab, cladribine, ocrelizumab, rituximab, mitoxantrone, azathioprine, cyclophosphamide	Total: 23, 3.5% Breast: 6, 26% GI: 5, 22% Gynecological: 5, 22% Endocrine: 2, 9% Prostate: 1, 4% Respiratory: 3, 13% Urinary: 1, 4%	3, 30%
Achiron (20)	2005	Israel	Retrospective cohort	1,338	-	67%	33%	RRMS: 776, 58% SPMS: 281, 21% PPMS: 107, 8% probable MS: 174, 13%	-	Glatiramer acetate, interferon beta, and IVig	Total: 15 Breast: 4, 26.7% Other female cancer: 7, 46.7% -“All male cancer”: 4, 26.7%	-
D’Amico (21)	2019	Italy	Retrospective cohort	1,180	32.8	67.1%	32.9%	RRMS: 854, 72.4% SPMS: 242, 20.5% PPMS: 84, 7.1%	4.2 (median)	Glatiramer acetate interferon beta, fingolimod, natalizumab, azathioprine, mitoxantrone and methotrexate	Total: 36, 3.1% Bladder: 8, 22.2% Thyroid: 6, 16.7% Colon: 6, 16.7% CNS: 5, 13.9% Lymphoma: 5, 13.9% Breast: 4, 11.1% Melanoma: 2, 5.6%	-
Lebrun (22)	2011	France	Retrospective cohort	20,993	RRMS: 32.4 SPMS: 42.9 PPMS: 42.1	72.5%	27.5%	RRMS: 13793, 65.7% SPMS: 5269, 25.1% PPMS: 1931, 9.2%	-	Interferon beta-1a, -1b, and glatiramer acetate, azathioprine, cyclophosphamide, mitoxantrone, mycophenolate mofetil, natalizumab, methotrexate, fingolimod, cladribine, and teriflunomide.	Total: 182, 0.9% Breast: 74, 40.7% Gynecological (uterine/ovarian): 28, 15.4% Urological (bladder/urethral/kidney): 10, 5.5% CNS: 11, 6% Prostate: 5, 2.7% Endocrine (Thyroid/adrenals/pituitary): 4, 2.2% Hematological (HL/NHL/leukemias): 11, 6% GI (Colon/rectal): 12, 6.6% Skin (Melanoma = 10; non-melanoma = 9): 19, 10.4% Lung: 5, 2.7% ENT: 3, 1.6%	-

Among the DMTs used, interferon beta (70%) and ocrelizumab (60%) were the most frequently associated with subsequent cancer diagnoses. The duration of DMT usage varied significantly (4 months to 30 years), with a median of 52 months. This wide range suggests that some malignancies may develop relatively soon after initiating therapy, which may be associated with rapidly growing tumors or lack of correlation with therapy altogether. However, others may take longer durations to manifest.

The types of cancers observed were diverse, with no single type predominating; however, gynecological tumors, including breast, were the most encountered malignancies, similar to the studies included in the literature review - albeit no skin cancers were found in our analysis. This variability indicates that the immunosuppressive effects of DMTs might not predispose patients to a specific type of cancer but rather increase overall susceptibility. Notably, six out of the ten cases were diagnosed at late stages (stage III or IV), emphasizing the need for regular cancer screening and early detection strategies in this patient population.

Our findings are consistent with previous studies that have reported an increased incidence of cancers associated with certain DMTs. For instance, the retrospective cohort study by Alping et al. (14) reported a cancer incidence of 2.7% among MS patients treated with rituximab, fingolimod, and natalizumab, with breast cancer being the most common malignancy (14). Similarly, Kingwell et al. (15) identified a 4.4% cancer incidence in patients treated with interferon beta, predominantly involving breast, lung, and prostate cancers (15) – but the latter two were not reported in our investigation. In Gomez-Moreno’s et al.’s prospective study which investigated dimethyl-fumarate found all their cancer cases to be female (16), in similarity to our findings which had an 80% female predominance. Our study presents a lower overall incidence, which may be attributed to differences in study design, population demographics, or the profile of DMTs utilized. Additionally, the shorter follow-up period in our study could contribute to an underestimation of the true long-term cancer risk.

The implications of our findings are multifaceted. First, they highlight the importance of personalized risk assessment when selecting DMTs for MS patients. Clinicians should consider the potential long-term risks of malignancy alongside the therapeutic benefits of these treatments, hence risk–benefit analyses regarding DMTs and associated malignancies are much needed in future studies. Second, our data support the need for regular and comprehensive cancer screening protocols for MS patients on DMTs, particularly for those on long-term therapy.

Given the observed variability in the types and stages of malignancies, a broad-based screening approach may be more beneficial than targeted screenings for specific cancers. Lastly, patient education on the potential risks and early signs of cancer should be a fundamental part of MS management.

Several limitations of our study must be acknowledged. The retrospective design and reliance on medical records may introduce selection and information biases. Additionally, the relatively small sample size and the inclusion of patients from only two centers may limit the generalizability of our findings to the general population of MS patients.

Future research should aim to include larger, multi-center cohorts with longer follow-up periods to better understand the long-term cancer risks associated with different DMTs. Prospective studies could provide more robust data and help identify specific patient subgroups at higher risk, facilitating more tailored screening and prevention strategies.

## Conclusion

Our study adds to the growing body of evidence suggesting a potential association between DMTs for MS and an increased risk of secondary malignancies. While the absolute risk appears relatively low, the diversity and severity of observed cancers underscore the need for ongoing vigilant screening. And due to the fact that half of the cases were late-stage cancers, we need to have a more robust and practical implementation of simple screening studies. By integrating regular cancer screening and patient education into routine MS care, clinicians can help diminish these risks and improve long-term outcomes for their patients.

## Data Availability

The original contributions presented in the study are included in the article/supplementary material, further inquiries can be directed to the corresponding author.

## References

[1] Compston A Coles A. Multiple sclerosis. Lancet. (2008) 372:1502–17. doi: 10.1016/S0140-6736(08)61620-7 18970977

[2] O’Gorman C Lucas R Taylor B. Environmental risk factors for multiple sclerosis: a review with a focus on molecular mechanisms. Int J Mol Sci. (2012) 13:11718–52. doi: 10.3390/ijms130911718, PMID: 23109880 PMC3472772

[3] McGinley MP Goldschmidt CH Rae-Grant AD. Diagnosis and treatment of multiple sclerosis. JAMA. (2021) 325:765–79. doi: 10.1001/jama.2020.26858 33620411

[4] Thompson AJ Banwell BL Barkhof F Carroll WM Coetzee T Comi G. Diagnosis of multiple sclerosis: 2017 revisions of the McDonald criteria. Lancet Neurol. (2018) 17:162–73. doi: 10.1016/S1474-4422(17)30470-2 29275977

[5] Correale J Gaitán MI Ysrraelit MC Fiol MP. Progressive multiple sclerosis: from pathogenic mechanisms to treatment. Brain. (2017) 140:527–46. doi: 10.1093/brain/aww258, PMID: 27794524

[6] Dargahi N Katsara M Tselios T Androutsou M-E De Courten M Matsoukas J. Multiple sclerosis: immunopathology and treatment update. Brain Sci. (2017) 7:78. doi: 10.3390/brainsci7070078, PMID: 28686222 PMC5532591

[7] Subei AM Cohen JA. Sphingosine 1-phosphate receptor modulators in multiple sclerosis. CNS Drugs. (2015) 29:565–75. doi: 10.1007/s40263-015-0261-z, PMID: 26239599 PMC4554772

[8] McGinley MP Cohen JA. Sphingosine 1-phosphate receptor modulators in multiple sclerosis and other conditions. Lancet. (2021) 398:1184–94. doi: 10.1016/S0140-6736(21)00244-0, PMID: 34175020

[9] Sellebjerg F Blinkenberg M Sorensen PS. Anti-CD20 monoclonal antibodies for relapsing and progressive multiple sclerosis. CNS Drugs. (2020) 34:269–80. doi: 10.1007/s40263-020-00704-w 31994023

[10] Zingaropoli MA Pasculli P Iannetta M Perri V Tartaglia M Crisafulli SG. Infectious risk in multiple sclerosis patients treated with disease- modifying therapies: a three-year observational cohort study. Mult Scler J Exp Transl Clin. (2022) 8:20552173211065731. doi: 10.1177/20552173211065731, PMID: 35003758 PMC8733376

[11] Lebrun C Rocher F. Cancer risk in patients with multiple sclerosis: potential impact of disease-modifying drugs. CNS Drugs. (2018) 32:939–49. doi: 10.1007/s40263-018-0564-y, PMID: 30143945

[12] Marrie RA Reider N Cohen J Stuve O Trojano M Sorensen PS. A systematic review of the incidence and prevalence of cancer in multiple sclerosis. Mult Scler. (2015) 21:294–304. doi: 10.1177/1352458514564489, PMID: 25533302 PMC4429168

[13] Cancer Incidence Report Saudi Arabia. (2020). Cancer incidence report Saudi Arabia. Available online at: https://shc.gov.sa/sites/English/Arabic/NCC/Activities/AnnualReports/Cancer%20Incidence%20Report%202020.pdf

[14] Alping P Askling J Burman J Fink K Fogdell-Hahn A Gunnarsson M. Cancer risk for fingolimod, natalizumab, and rituximab in multiple sclerosis patients. Ann Neurol. (2020) 87:688–99. doi: 10.1002/ana.25701 32056253

[15] Kingwell E Evans C Zhu F Oger J Hashimoto S Tremlett H. Assessment of cancer risk with β-interferon treatment for multiple sclerosis. J Neurol Neurosurg Psychiatry. (2014) 85:1096–102. doi: 10.1136/jnnp-2013-307238, PMID: 24594506

[16] Gómez-Moreno M Sánchez-Seco VG Moreno-García S Cámara PS Sabin-Muñoz J Ayuso-Peralta L. Cancer diagnosis in a Spanish cohort of multiple sclerosis patients under dimethylfumarate treatment. Mult Scler Relat Disord. (2021) 49:102747. doi: 10.1016/j.msard.2021.102747, PMID: 33524928

[17] Ragonese P Aridon P Vazzoler G Mazzola MA Lo Re V Lo Re M. Association between multiple sclerosis, cancer risk, and immunosuppressant treatment: a cohort study. BMC Neurol. (2017) 17:155–6. doi: 10.1186/s12883-017-0932-0, PMID: 28789625 PMC5549380

[18] Nørgaard M Veres K Didden EM Wormser D Magyari M. Multiple sclerosis and cancer incidence: a Danish nationwide cohort study. Mult Scler Relat Disord. (2019) 28:81–5. doi: 10.1016/j.msard.2018.12.014 30576846

[19] Mariottini A Forci B Gualdani E Romoli M Repice AM Barilaro A. Incidence of malignant neoplasms and mortality in people affected by multiple sclerosis in the epoch of disease-modifying treatments: a population-based study on Tuscan residents. Mult Scler Relat Disord. (2022) 60:103679. doi: 10.1016/j.msard.2022.103679, PMID: 35217486

[20] Achiron A Barak Y Gail M Mandel M Pee D Ayyagari R. Cancer incidence in multiple sclerosis and effects of immunomodulatory treatments. Breast Cancer Res Treat. (2005) 89:265–70. doi: 10.1007/s10549-004-2229-4, PMID: 15754125

[21] D’Amico E Chisari CG Arena S Zanghì A Toscano S Lo Fermo S. Cancer risk and multiple sclerosis: evidence from a large Italian cohort. Front Neurol. (2019) 10:337. doi: 10.3389/fneur.2019.00337, PMID: 31024431 PMC6469363

[22] Lebrun C Vermersch P Brassat D Defer G Rumbach L Clavelou P. Cancer and multiple sclerosis in the era of disease-modifying treatments. J Neurol. (2011) 258:1304–11. doi: 10.1007/s00415-011-5929-9, PMID: 21293872

